# Effectiveness of dry cow therapy and/or internal teat sealant on existing infections in smallholder dairy farms in Kenya

**DOI:** 10.14202/vetworld.2021.1430-1436

**Published:** 2021-06-04

**Authors:** Ronald K. Sang, George K. Gitau, John A. Van Leeuwen

**Affiliations:** 1 Department of Clinical Studies, University of Nairobi, Nairobi, Kenya; 2 Department of Health Management, University of Prince Edward Island, Canada

**Keywords:** dairy, dry cow therapy, effectiveness, mastitis, smallholder

## Abstract

**Background and Aim:**

Dry cow therapy (DCT) can be an effective treatment of mastitis that has not responded to conventional treatment during lactation. The aim of this study was to establish the effectiveness of DCT options available in reducing intramammary infections in smallholder dairy farms in Kiambu County, Kenya.

**Materials and Methods:**

The study targeted smallholder dairy farms which were registered at the local dairy cooperatives and which had cows that were at the point of dry-off. A total of 32 cows with 121 quarters that were California Mastitis Test (CMT) positive were recruited, with the quarters randomly allocated to receive either DCT (DCT – neomycin sulfate, penethamate hydriodide, and procaine benzylpenicillin) and internal teat sealant (ITS) or ITS alone (bismuth nitrate) after aseptically collecting quarter milk samples for bacterial culture. Farm- and animal-level factors were captured through a questionnaire which was administered to the principal farmer or a person who was managing the animals. Post-calving, milk samples were also collected for bacterial culture to establish if the infection was cleared or if there was a new infection.

**Results:**

DCT with ITS significantly reduced the proportion of quarters infected with *Staphylococcus aureus* from 64.0% at dry-off to 44.0% post-calving (35% reduction). In the control group, ITS alone, there was a small reduction in proportions of *S. aureus* from 46.8% to 40.4%. Proportions of quarter infections by coagulase-negative *Staphylococcus* in the treatment group reduced from 16.0% at dry-off to 2.0% post-calving, with a significant reduction in the control group too from 19.1% to 4.3%, which could be due to self-cure. *Actinomyces* species, *Escherichia coli*, *Streptococcus* species, and *Pseudomonas* species proportions slightly increased in the treatment group, as did *E*. coli and *Pseudomonas* species proportions in the control group.

**Conclusion:**

In smallholder dairy farms with subclinical mastitis, DCT of CMT-positive cows leads to a significant decrease of *S. aureus* infections at calving.

## Introduction

Mastitis continues to be a major constraint in the dairy industry worldwide and is associated with economic losses and changes in the udder [1-3]. There are several prevention and control practices that have been used in curbing the disease in dairy farms. Dry cow therapy (DCT) is one of the prevention and control methods and is key in the treatment of mastitis that has not responded to conventional treatment during lactation, such as cases of *Staphylococcus aureus*; thus, use of DCT is advised, especially if the case is chronic [4]. There are two reasons why DCT is more effective in treating mastitis than antibiotic treatment during lactation: (1) The DCT intramammary tube has a higher concentration of antibiotic than a lactation intramammary tube; and (2) at dry-off, the antibiotic is no longer removed with the subsequent milking. These two reasons ensure sustained high concentration of antibiotics in the udder tissues at the start of the dry period. The slow metabolism of the DCT during the early dry period also prevents new intramammary infections while the keratin plug is being formed in the teat sphincter [5]. Furthermore, since the high doses of antibiotics can be used without concerns for milk withdrawal, DCT provides prolonged activity of the drug for high prevention and cure rates with minimal chance of residues in milk [6].

There are two main approaches that have been used in the practice of DCT in the dairy industry; blanket and selective DCT. Blanket DCT involves treatment of all quarters at dry-off without evidence of intramammary infections, while selective DCT is treatment of quarters that are test-positive for intramammary infections. Blanket DCT has been the preferred DCT method among dairy farmers in North America and most parts of the world for decades, but with the movement against increased antimicrobial resistance, selective DCT is becoming more common [7,8]. Blanket DCT is an expensive approach in the management of intramammary infections because every quarter of every cow is treated at dry-off, but it can be helpful on farms with increased prevalence of mastitis during lactation and increased incidence of dry-cow mastitis but low antimicrobial resistance [9]. Conversely, selective DCT is more economical when bulk somatic cell count and both prevalence and incidence of mastitis are low. Therefore, the appropriate approach for a farm depends on its risks of mastitis and the restrictions in use of antibiotics [8].

Apart from the use of antibiotics, there are also other approaches to the prevention of new infections during the dry period, such as the use of teat sealants. Naturally, within 2 weeks after dry-off, the keratin plug forms in the teat sphincter to act as a barrier to prevent the entry of harmful organisms [10]. However, the formation of the plug may fail in some cows and the integrity of the plug decreases as calving approaches; therefore, some cows can still be prone to new intramammary infections during the dry period [11]. Teat sealants act as the natural barrier and are applied at dry-off. There are two types of teat sealants that are being used in the dairy industry; internal and external teat sealants. Crispie *et al*. [10] found that external teat sealants are not as effective as internal teat sealants (ITS), but offer some protection as compared to unsealed teats. Teat sealants can be used in combination with antibiotic DCT, and the continued worry of antimicrobial resistance has led to the increased usage of teat sealants alone in selective DCT programs [10].

In Europe, Mutze *et al*. [11] and Newton *et al.*, [12] reported that incidences of mastitis post-calving in cows treated with DCT and ITS were lower compared to those treated with DCT alone or with no treatment at all. However, there is limited research on ITS and DCT on smallholder dairy farms in developing countries. ITS, when used alone or together with DCT, have been shown to reduce mastitis post-calving by 75% [13] as compared with untreated cows on large commercial farms in Kenya, noting that the combination of ITS and DCT significantly reduced clinical mastitis caused by environmental pathogens. However, more research is required to determine if similar results would occur in smallholder Kenyan farms.

The objectives of this study were to determine: (1) The types of bacterial infections in dairy cows at dry-off using the California Mastitis Test (CMT) and culture and (2) the effectiveness of different DCT options for treating existing mastitis infections at dry-off.

## Materials and Methods

### Ethical approval and informed consent

The study was approved by Biosecurity, Animal Use and Ethics Committee, Faculty of Veterinary Medicine, University of Nairobi (FVM BAUEC/2020/258). The research was done according to the local laws and guidelines by International Animal Ethics Committee or Institutional Ethics Committee. The details of the study were discussed with the owners of animals targeted for recruitment. They were free to participate or withdraw from the study.

### Study period and area

**Figure-1:**
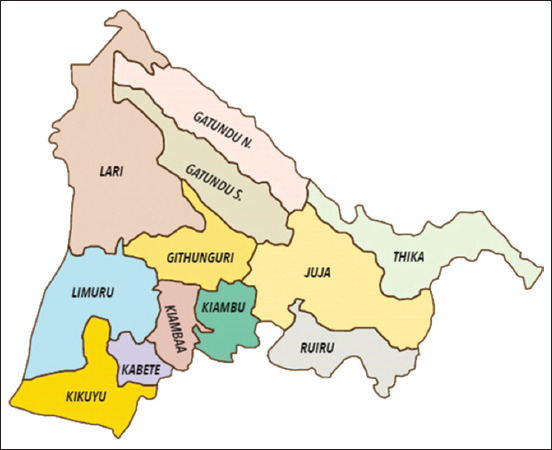
Geographical location of Kikuyu sub-county in Kiambu County [Source: https://kiambu.go.ke/political-units/].

The study was carried out from July 2019 to March 2020 in Kikuyu Sub-County of Kiambu County in the central part of Kenya (Figure-1). The county experiences a warm climate with night-time temperatures ranging between 12°C and 18.7°C, and day-time temperatures ranging between 15°C and 26.7°C. Annual rainfall averages 1000 mm, which makes the county suitable for agriculture. Crop production and livestock-keeping are the major economic activities in the area due to the ready market access in nearby Nairobi City. Dairy farming contributes immensely to the economy of the Kiambu area, and this is evident by the presence of milk-processing plants in the area, such as Brookside, Palmside, Githunguri, and Ndumberi Dairies, among others.

Kiambu County was conveniently selected for the study for various reasons: (1) There is intensive smallholder dairy farming in the county, **(**2) there was another ongoing project among its smallholder dairy farms that allowed for efficient research funding utilization through shared transportation costs, and **(**3) it had close proximity to the Department of Clinical Studies Laboratory, allowing reliable milk culture procedures without compromising the quality of the milk samples collected.

### Study design and selection of farms

The study was a randomized controlled trial that was targeting cows to be dried off. Inclusion criteria for eligible farmers for the study included: (1) Farmers who had a cow that was being dried off and (2) farmers who were actively delivering milk to the Kabete and Muguga Dairy Cooperative Societies. Therefore, farms were conveniently selected based on the availability of a cow at the point of drying off. Inclusion criteria for eligible cows for the study included cows that tested positive for CMT on at least one quarter at dry-off on the eligible farms.

The sample size for the treatment part of the study was 100 positive quarters in total and was estimated using a DCT cure rate of 70% in the treatment group and 40% in the control group, as reported in the Netherlands [14]. This sample size was computed based on a power of 0.8 and a significance level of 0.05.

### Data, sample collection, and allocation of treatment

Selected farms were visited from July 2019 to March 2020 where questionnaires were administered to the principal farmers to capture data on farm- and animal-level factors. Farm-level factors of interest included farmer demographics, cow housing management, milking practices, and reported past diseases, such as mastitis, and their control, among others. Animal-level factors were breed of cow, age, parity, current level of production, and month of lactation. Quarter-level factors included current and past mastitis status, current and past milk leakage status, and current teat-end status, among others.

In addition, CMT was done on the cows to be dried off, and milk samples were collected for bacterial culture from quarters with CMT score 2 and 3. Approximately 5 ml of milk was aseptically collected from each positive quarter into separate sample bottles which were stored in an ice-packed cool box for transportation to the Department of Clinical Studies bacteriology laboratory for bacterial culture.

The CMT-positive quarters were then randomly allocated to receive either DCT (Neomycin sulfate 100 mg, penethamate hydriodide 100 mg, and procaine benzylpenicillin 400 mg per 4.5 g syringe) and ITS (65% bismuth subnitrate) or ITS only by the research team. The randomization was achieved by randomly allocating a number to each quarter by the lead author so that quarter number 1 received the treatment while quarter number two received the control.

Within the 1^st^ week after calving, the study cows were again examined, CMT administered, and samples taken from quarters with CMT >1. Some farms were visited a 3^rd^ time 2 weeks after calving to confirm post-calving culture-negative status with a CMT test, and samples again taken from quarters with CMT >1.

### Laboratory culture

Approximately 10 mL of milk were streaked on blood and MacConkey agar plates and incubated at 37°C for 48 h, with the remaining milk being frozen at −20°C. Growth was observed, and in samples that showed no growth, the frozen milk samples were cultured to check for the presence of *S. aureus* since these bacteria are known to be released from lysed milk lymphocytes post-freezing. Colonies were described and Gram-staining and biochemical tests were used to characterize the organisms, such as the coagulase test. This coagulase test was used to differentiate *S. aureus* from coagulase-negative *Staphylococci* such as *Staphylococcus* *epidermidis* and *Staphylococcus* *saprophyticus*.

### Data entry and analysis

Data collected using the questionnaires, along with the results from the laboratory, were entered into Microsoft Excel (Microsoft Inc., Sacramento, California, USA) where they were cross-checked for accuracy and coded, before being imported to Stata 15.1 (StataCorp LLC, College station, Texas, USA) for analyses. Proportions (presented as percentages) were determined for categorical variables, such as breed, while means, ranges, and standard deviations were determined for continuous variables, such as age. To determine if the intervention of DCT and ITS was significantly different from the ITS only at dry-off, the proportions of infected quarters at dry-off were compared to the proportions of infected quarters post-calving, for all types of infections, and by bacteria. This comparison was done by testing if there was a significant interaction between time of sampling and treatment group when stratifying the data and using infection as the outcome of interest.

## Results

This study involved 20 smallholder dairy farms and 32 dairy cows with a total of 121 CMT-positive quarters. During the first visit (dry-off), all 121 quarters were sampled. During the second visit, only 97 samples were collected and cultured because three cows were lost during the study, two through disposal following Downer cow syndrome at calving and one through sale. Another 12 samples from three other cows could not be processed in time following disruption of normal laboratory functions due to COVID-19, leading to 26 cows being cultured post-calving. No side effects in the study cows were reported by the farmers or noticed by the researchers.

### Farm and animal demographics and management

This section provides a description of the study farmers and farms so the results can be interpreted in the context of these farm characteristics. The mean age of the participating farmers was 51 years, and the mean number of years in the dairy business was 15 years. The mean number of milking cows, dry cows, heifers, calves, and bulls was 4, 1, 3, 2, and 0.3, respectively, with the mean herd size being 11 animals. Mean average daily milk production for the dairy farms was 55 L, with dairy contributing to an estimated mean percentage income of 38% (Table-1).

**Table-1:** Descriptive statistics of continuous variables from 20 smallholder farms in Kiambu County, Kenya, from September 2019 to March 2020.

Variable	Mean	Range	Standard deviation
Primary farmer age (years)	51.0	27-75	13.9
Number of adults in household	3.9	1-12	2.3
Number of years in dairy farming	15.2	6-40	9.0
Farm acreage (acres)	0.67	0.125-3.0	0.77
Number of dry cows	1.3	0-4	1.1
Number of milking cows	4.2	1-13	3.1
Number of heifers	2.9	0-6	2.0
Number of calves	2.3	0-11	2.6
Number of bulls	0.3	0-4	0.92
Herd size	10.9	3-34	6.9
Average milk production per day (kg)	54.7	1-200	53.1
Percentage income from dairy	37.5	5-100	30.2
Number of cubicles in animal housing	8.4	2-15	4.1

The results further showed that 70% of the farmers interviewed were male, while 30% were female. Of all the farmers interviewed, 50% had attained tertiary education, with 15% and 35% having attained primary and secondary education, respectively. In addition, 60% of the farms were under the management of husbands, followed by wives (25%), children (10%), and employees (5%).

Most farms (75%) used hand-milking, and of these, 47% squeezed (instead of pulling) the teats during milking, while 15% used machine milking and 10% used both methods (Table-2). Milking 2 times a day was practiced by 80% of the farms, with 20% milking 3 times a day. In addition, all the farms used cloth for udder cleaning, with 90% using one cloth for all the cows in the farm and 95% washing and drying the cloth between milking times. Teat dipping post-milking was being done by only 15% of the recruited farms, while pre-milking jelly was used by 80% of the farms. There were 44% of cows being milked twice daily, and 28% each being milked once and thrice daily, with 56% having no history of mastitis. In addition, 80% of the farmers dried off their cows gradually over 14 days, with 40% reporting that they sometimes used DCT (Table-2).

**Table-2:** Descriptive statistics on milking practices from 20 smallholder dairy farms in Kiambu County, Kenya, from September 2019 to March 2020.

Variable	Category	Frequency	Percentage
Milking method	Hand	15	75
	Machine	3	15
	Both	2	10
Hand milking method (n=17 farms)	Squeeze	8	47
	Pull	9	53
Feeding method	Zero-grazing	19	95
	Open-grazing	0	0
	Both	1	5
Pre-milking udder cleaning	No	0	0
	Yes	20	100
Temperature of water used for udder cleaning	Warm	19	95
	Cold	1	5
Addition of disinfectant into water for udder cleaning	No	17	85
	Yes	3	15
Udder drying before milking	No	2	10
	Yes	18	90
Type of material used for udder drying	Cloth	18	90
	None	2	10
Use of separate cloth per cow	No	18	90
	Yes	2	10
Washing hands before milking	No	0	0
	Yes	20	100
Cloth washing between milking	No	1	5
	Yes	19	95
Cloth drying between milking	No	1	5
	Yes	19	95
Use of teat dip post-milking	No	17	85
	Yes	3	15
Use of milking jelly pre-milking	No	4	20
	Yes	16	80
Feeding immediately after milking	No	5	25
	Yes	15	75
Number of milkings per day	2×	16	80
	3×	4	20
History of mastitis in the last 1 year	No	3	15
	Yes	17	85
Management of mastitis in the last year (n=17 farms)	Treated	16	94
	Sampled and treated	1	6
Person who treats mastitis	Farmer	2	10
	AHA^1^	17	85
	Veterinarian	1	5
Method of drying cows	Abrupt	4	20
	Gradual	16	80
Use of dry cow therapy	No	12	60
	Yes	8	40
Blanket dry cow therapy	No	14	70
	Yes	6	30
Use of teat sealants at dry-off	No	20	100
	Yes	0	0

1 Animal Health Assistant

The study also showed that 70% of the farmers reported that clots in milk were a sign of mastitis in cows, while 45% reported swollen udder and 25% reported pain during milking. Most (85%) of the farmers said that mastitis lasted for 1 week following treatment, with 90% saying that mastitis resulted in decreased milk production (Table-3).

**Table-3:** Descriptive statistics on farmer perceptions on mastitis from 20 smallholder dairy farms in Kiambu County, Kenya, from September 2019 to March 2020.

Variable	Category	Frequency	Proportion (%)
Swollen udder as a sign of mastitis	No	11	55
	Yes	9	45
Abnormal milk as a sign of mastitis	No	16	80
	Yes	4	20
Clots in milk as a sign of mastitis	No	6	30
	Yes	14	70
Reduced milk as a sign of mastitis	No	17	85
	Yes	3	15
Udder fibrosis as a sign of mastitis	No	17	85
	Yes	3	15
Fever as a sign of mastitis	No	19	95
	Yes	1	5
Pain as a sign of mastitis	No	15	75
	Yes	5	25
Impact of mastitis on milk production	Decreased	18	90
	Constant	1	5
	Increased	1	5
Duration of mastitis	2 days	1	5
	1 week	17	85
	Above 1 week	2	10

### Bacterial isolates at dry-off and post-calving

In Table-4, proportions of bacteria isolated at the cow level from the 20 smallholder dairy farms in Kiambu County at dry-off (n=26) and post-calving (n=26). *S. aureus* were isolated from at least one quarter in 80.8% of the study cows with positive CMT in at least one quarter at dry-off, and this proportion dropped to 65% post-calving. Coagulase-negative *Staphylococcus* was the next highest pathogen identified at dry-off, at 34.6% of CMT-positive cows, but this proportion dropped to 11% post-calving. Other pathogens were cultured in low proportions <20% at dry-off and post-calving.

**Table-4:** Proportions of bacteria isolated at the cow level from 20 smallholder dairy farms in Kiambu County at dry-off (n=26) and post-calving (n=26), from September 2019 to March 2020.

Variable	Category	Frequency	Proportion	Proportion difference
*Staphylococcus aureus*	Dry-off	21	80.8	
	Post-calving	17	65.4	−15.4
*Actinomyces* species	Dry-off	2	7.7	
	Post-calving	1	3.9	−3.8
Coagulase-negative *Staphylococcus*	Dry-off	9	34.6	
	Post-calving	3	11.5	−23.1
*Escherichia coli*	Dry-off	1	3.8	
	Post-calving	5	19.2	+15.4
*Pseudomonas* species	Dry-off	1	3.8	
	Post-calving	2	7.7	+3.9
*Streptococcus* species	Dry-off	3	11.5	
	Post-calving	2	7.7	−3.8

The trial results showed that the combination of DCT and ITS significantly reduced the proportions of *S. aureus* quarter infections from 64.0% at dry-off to 44.0% post-calving, while the quarter-level infections of *S. aureus* in the control group (ITS only) only reduced from 46.8% at dry-off to 40.4% post-calving (Table-5). The treatment group reductions were substantial considering the difficulty in eliminating cases of mastitis with *S. aureus*.

**Table-5:** Proportions of bacterial isolates at dry-off and post-calving, by treatment group and control group, in 97 quarters from 26 cows on 19 smallholder dairy farms in Kiambu County, Kenya, from September 2019 to March 2020.

Isolate	DCT+ITS		ITS only	
	
	Dry-off %	Post-calving %	Dry-off %	Post-calving %
*Staphylococcus aureus*	64.0 (32/50)	44.0* (22/50)	46.8 (22/47)	40.4 (19/47)
Coagulase-negative *Staphylococcus*	16.0 (8/50)	2.0* (1/50)	19.1 (9/47)	4.3* (2/47)
*Escherichia coli*	2.0 (1/50)	6.0 (3/50)	0 (0/47)	6.3 (3/47)
*Pseudomonas* Spp.	2.0 (1/50)	2.0 (1/50)	0 (0/47)	2.1 (1/47)
*Streptococcus* Spp.	0 (0/50)	4.0 (2/50)	6.4 (3/47)	0 (0/47)
*Actinomyces* Spp.	2.0 (1/50)	2.0 (1/50)	2.1 (1/47)	0 (0/47)

* Significant difference (p<0.05) between dry-off percent and post-calving percent within the pathogen group. ITS=Internal Teat Sealant, DCT=Dry cow therapy

Coagulase-negative *Staphylococcus* infections in the intervention group at the quarter-level lowered from 16.0% at dry-off to 2.0% post-calving. However, there was also a significant reduction in coagulase-negative *Staphylococcus* infections in the control group from 19.1% at dry-off to 4.3% post-calving.

The results further showed that there was a stable or slight increase in proportions of *Escherichia coli*, *Streptococcus, Pseudomonas*, and *Actinomyces* species in the DCT and ITS group. The DCT+ITS group did seem to cure all the *Streptococcus* and *Actinomyces*. There was no *E. coli* or *Pseudomonas* species infections in the control group at dry-off, but there were a small number of these pathogens isolated post-calving in the control group, similar to the proportions in the intervention group. There were no significant differences between proportions of other pathogens in the two groups.

## Discussion

Treatment with dry cow intramammary antibiotics and ITS showed a significant reduction in the proportion of *S. aureus* isolates from 64.0% of CMT-positive quarters at dry-off to 44.0% at post-calving (31.3% cure). This result was in agreement with findings by Mutze *et al*. [11] who reported that there was a reduction of prevalence by 25-75% in the first 100 days of lactation in quarters that received ITS and a long-acting antibiotic. However, our cure rate for *S*. aureus was low compared to the cure rate of 77% reported in a meta-analysis by Halasa *et al*. [15]; this higher cure rate was for all *Staphylococcus* species, not just *S. aureus*. The *S. aureus* organisms develop micro-abscesses around themselves, thus making treatment difficult since antibiotics cannot penetrate the micro-abscesses without ample concentration and duration of exposure, especially if the infection is long-standing, leading to thick walls of the micro-abscesses [4]. As a result, lactational and DCT have been reported to have lower efficiency against *S. aureus* as compared to Streptococcal bacteria for many years [15]. The high proportion of *S. aureus* infections in CMT-positive cows at dry-off in our study (80.8%), along with the substantial cure rate (31.3%) and low self-cure rate (13.6%) demonstrates the need for DCT utilization in smallholder dairy herds for CMT-positive cows.

In this study, the proportions of coagulase-negative *Staphylococcus* infections reduced from 16.0% to 2.0% in the intervention group, but there was also a significant reduction in coagulase-negative *Staphylococcus* infection in the control group from 19.1% to 4.3%, and these reductions could be attributed to the self-cure phenomenon. This self-cure phenomenon was in agreement with findings in various studies that received DCT + ITS [12,16].

The proportions of *E. coli*, *Pseudomonas* species, *Streptococcus* species, and *Actinomyces* species were stable or increased slightly in the treatment group; however, the proportion of *E. coli* and *Pseudomonas* species also increased slightly in the control group. These new infections could be explained by management practices during the dry period or post-calving period which might have exposed the quarters to new infections. Dry cow intramammary antibiotics are effective in management of existing intramammary infections and control of new infections in the early dry period but will not prevent new infections toward the end of the dry period due to reduction of antibiotic levels below the minimum inhibitory concentration [17,18]. Bradley *et al*. [16] also reported new intramammary cases of *E. coli* and other *Enterobacteriaceae* in quarters that had been given intramammary antibiotics at dry-off. In a meta-analysis study, it was reported that quarters that received DCT were 0.61 times less likely to develop new intramammary infections of all types of organisms (particularly *Staphylococcus* and *Streptococcus* species, but not *E. coli*) during the dry period as compared to untreated quarters [18].

In the control group, there was a slight drop in the proportion of *S. aureus* infections from 46.8% to 40.4%, *Streptococcus* species from 6.4% to 0, and *Actinomyces* species from 2.1% to 0. These reductions could be attributed to self-cure, particularly the *Streptococcus* species, as Huxley *et al*. [19] also reported cure rates in quarters that received teat sealants only. Halasa *et al*. [18] also reported spontaneous average cure rates of 46% and 52% in two control groups of untreated quarters in a meta-analysis.

The study has some limitations that include lack of antimicrobial sensitivity results, which occurred because COVID-19 shut down the bacteriology lab during the planned time for sensitivity analyses. Furthermore, it would have also been very helpful to carry out PCR on the isolated organisms to determine if the strains at dry-off were the same as those post-calving, differentiating unsuccessful treatment from new infections. Farmers and field researchers were not blinded to treatments (two tubes vs. one tube), but the laboratory staff were blinded, and they provided the key outcome of culture results. The relatively small number of herds and cows in this study could lead to questionable representativeness of the herds and their descriptive results, and lower power to detect significant cure rates.

Future research should include a determination of the prevalence of subclinical mastitis at dry-off on smallholder dairy farms in the tropics. Furthermore, the efficacy of DCT to treat mastitis at dry-off on smallholder dairy farms should include antimicrobial sensitivity and molecular analysis of the isolates to understand better the sensitivity profiles and development of new infections during the dry period.

## Conclusion

On smallholder dairy farms with subclinical mastitis, DCT of CMT-positive cows using DCT (Neomycin sulfate 100mg, penethamate hydriodide 100 mg, and procaine benzylpenicillin 400 mg per 4.5 g syringe) and ITS (65% bismuth nitrate) leads to a significant decrease of the mastitis infections post-calving.

## Authors’ Contributions

RKS: Collected the data and drafted the manuscript, GKG: Did data analysis and proofread the manuscript. JAVL: Designed the study, acquired research funds and proofread the manuscript. All authors read and approved the final manuscript.
